# First molecular identification of *Spirometra mansoni* in the golden jackal (*Canis aureus*) in Croatia

**DOI:** 10.3389/fvets.2025.1629099

**Published:** 2025-08-11

**Authors:** Ana Šikić, Ema Gagović, Alicia Rojas, Magda Sindičić, Daria Jurković Žilić, Šimun Naletlić, Davor Balić, Adnan Hodžić, Relja Beck

**Affiliations:** ^1^Department for Parasitology and Invasive Diseases, Faculty of Veterinary Medicine, University of Zagreb, Zagreb, Croatia; ^2^Laboratory for Parasitology, Department of Microbiology and Parasitology, Croatian Veterinary Institute, Zagreb, Croatia; ^3^Laboratory of Helminthology, Faculty of Microbiology, University of Costa Rica, San José, Costa Rica; ^4^Department for Hunting and Wild Animals, Faculty of Veterinary Medicine, University of Zagreb, Zagreb, Croatia; ^5^Laboratory for Pathology, Department of Pathology, Croatian Veterinary Institute, Zagreb, Croatia; ^6^Veterinary Department Vinkovci, Croatian Veterinary Institute, Vinkovci, Croatia; ^7^Center for Microbiology and Environmental Systems Science (CMESS), Department of Microbiology and Ecosystem Science, Division of Microbial Ecology (DoME), University of Vienna, Vienna, Austria

**Keywords:** *Canis aureus*, *cox1*, *nad1*, Croatia, *Spirometra mansoni*

## Abstract

This study presents the first molecularly confirmed identification of the cestode *Spirometra mansoni* in the golden jackals (*Canis aureus*) in Croatia, and possibly the first such report in Europe. Of 198 jackals examined between 2020 and 2025, adult *Spirometra* worms were recovered from three individuals. The morphological characteristics of these specimens were consistent with *S. mansoni*, and their identity was confirmed by PCR and sequencing of the mitochondrial *cox1* and *nad1* genes. Phylogenetic analysis grouped the obtained sequences within the *S. mansoni* clade, with strong posterior probability support. This finding expands the known host range and geographic distribution of *S. mansoni* and underscores the importance of integrating molecular diagnostics in parasitological surveys. Further research is needed to assess the role of golden jackals and other wildlife in the epidemiology of *Spirometra* spp. in Europe.

## Introduction

In recent years, parasites of the genus *Spirometra* (family Diphyllobothriidae, order Diphyllobothriidea) have increasingly attracted the attention of veterinary and human health professionals in Europe, primarily due to their role as causative agents of the food-borne zoonosis known as sparganosis. The disease was first described in China in 1882 (1, 2), and since then, more than 2,000 cases have been reported globally—predominantly in East and Southeast Asia—with sporadic cases documented in South America, Africa, and Europe (3, 4).

Despite the growing number of reports, many aspects of *Spirometra* biology remain unresolved, particularly in terms of taxonomy, geographical distribution, host range, zoonotic potential, and disease epidemiology (4–7). Adult *Spirometra* worms are morphologically similar to those within order Diphyllobothriidea, exhibiting considerable morphological uniformity as well as host-related intraspecific variability. Consequently, reliable identification often requires molecular methods, although there is still a lack of consistent morphological data linked to genetically confirmed specimens (7). Additionally, available molecular data for some *Spirometra* species have been inconsistent, with many nucleotide sequences available in the GenBank^®^ database being misidentified (7, 8). Therefore, identification within the genus should be supported by molecular, morphological, ecological, or pathological evidence, as previously suggested (9).

Molecular studies suggest the existence of at least seven genetically distinct lineages or species within the genus, as summarized in the comprehensive taxonomic reviews by Kuchta et al. (4, 7). These tapeworms have a complex life cycle involving two intermediate hosts. The first larval stage (coracidium) hatches from eggs in water and is ingested by copepods (*Cyclops* spp.), in which the procercoid develops. The second intermediate host—typically amphibians, reptiles, or mammals—ingests the copepod, after which the plerocercoid larva develops and migrates to the subcutaneous or muscular tissues. Carnivorous mammals serve as definitive hosts, becoming infected upon consuming the second intermediate host (10, 11).

*Spirometra* species have been reported from all continents except Antarctica. While *S. mansoni* (Cobbold, 1882) is considered cosmopolitan, other species appear to have more restricted distributions. However, due to the lack of molecular confirmation in most reports, current distribution maps remain unreliable and subject to revision (4, 7). In Europe, only two species, *S. erinaceieuropaei* and *S. mansoni* have been confirmed through molecular analysis (4). Plerocercoid stages have been identified morphologically in 17 European countries, but records of definitive hosts remain sparse. *Spirometra erinaceieuropaei* is potentially endemic to Central and Eastern Europe and exhibits low genetic diversity (12). Other historical species (*Spirometra ranarum, Spirometra raillieti*) are considered taxonomically doubtful due to insufficient descriptions and a lack of genetic data (7).

Although European wildlife harbors a wide array of potential definitive and intermediate hosts, there is a significant knowledge gap regarding their role in *Spirometra* transmission. The genus has been molecularly confirmed in only three definitive host species, namely wolves (*Canis lupus*), Eurasian lynxes (*Lynx lynx*), and domestic cats (*Felis catus*). Moreover, morphological records exist for the European wildcat (*Felis silvestris*), red fox (*Vulpes vulpes*), and raccoon dog (*Nyctereutes procyonoides*) (4, 7, 12, 13). Among all native European carnivores, the golden jackal (*Canis aureus*) is currently undergoing the most rapid and dramatic range expansion (14). This medium-sized, omnivorous and opportunistic canid is distributed across southern Asia, the Middle East, and outheastern to central Europe, where it inhabits a wide range of habitats, including agricultural landscapes and semi-urban areas (15, 16). Golden jackals are highly adaptable to human-modified habitats, where they readily exploit accessible food sources (17). Due to this ecological flexibility, they have emerged as one of the most relevant species for investigating the role of potential definitive hosts of Spirometra in Europe. This study presents the first molecular identification of *Spirometra mansoni* in golden jackals in Croatia, contributing to a broader understanding of the parasite's host range and geographic distribution.

## Materials and methods

### Study area

This study was conducted in Central Croatia, a region situated in the continental part of the country that features lowland and hilly landscapes interspersed with river valleys and forested zones. The sampling locations were located within a mosaic of mixed deciduous forests, agricultural land, shrub vegetation, and rural settlements, typical of the transitional zone between the Pannonian Basin and the Dinaric Mountains. The climate is temperate continental, characterized by hot summers, cold winters, and significant seasonal variations in both temperature and precipitation (18). The region is rich in freshwater resources, with numerous rivers, streams, and wetlands distributed throughout the area. These water bodies, along with extensive forest coverage, provide diverse habitats for a variety of amphibians, reptiles, birds, and mammals (19).

The ecological heterogeneity and the presence of permanent surface water make this landscape particularly favorable for supporting complex food webs and parasite transmission cycles. Specifically, the proximity to natural water sources, forest edges, and agricultural fields creates a dynamic interface suitable for the coexistence of both intermediate and definitive hosts of various parasitic species. The golden jackal is well adapted to such environments and frequently occupies ecotones between natural and anthropogenic habitats.

### Parasite collection and morphological identification

A total of 198 golden jackal carcasses were submitted to the Croatian Veterinary Institute between 2020 and 2025. The carcasses were collected either as part of the national rabies surveillance program or through roadkill monitoring. The approximate geographic origin was recorded for each animal. For better visibility of the obtained results, positive samples were mapped using QGIS software version 3.30.0 RC. During the necropsy, strict biosafety measures were implemented to prevent potential infection with *Echinococcus* spp. The intestines were opened longitudinally, and their contents visually inspected. Macroscopically visible tapeworms, were isolated, washed in water, and preserved in 70% ethanol. Prior to preservation specimens were morphologically identified using available morphological keys (7) under a Zeiss Stereo Discovery V20 and Imager M.2 microscope, equipped with Axiovision and ZEN2 Pro software.

### Molecular characterization and phylogenetic analysis

Genomic DNA was extracted from individual worms from each animal using the DNeasy Blood and Tissue Kit (Qiagen, Germany) on the QIAcube robotic workstation (Qiagen, Germany). PCR amplification of the mitochondrial *cytochrome c oxidase subunit* 1 (*cox1*) gene was performed using primers Diphyllo-Cox1-F (5′-TAGACTAAGTGTTTTCAAAACACTA−3′) and Diphyllo-Cox1-R (5′-ATAGCATGATGCAAAAGG−3′) (20), while the *NADH dehydrogenase subunit* 1 (*nad1*) gene was amplified using primers JB11 and JB12 (21). PCR amplification was conducted with GoTaq^®^ G2 Hot Start Colorless Master Mix (Promega, USA) under the following cycling conditions: 40 cycles of 30 s at 94°C, 30 s at 56°C (*cox1*) or 50°C (*nad1*), and 60 s at 72°C.

Amplicons were verified by capillary electrophoresis (QIAxcel, Qiagen, Germany), purified using ExoSAP-IT^®^ PCR Clean-Up Reagent (Thermo Fischer Scientific, USA), and sequenced bidirectionally by Macrogen Europe (macrogen-europe.com). Obtained nucleotide sequences were then aligned and analyzed with SeqMan and EditSeq (Lasergene, DNASTAR) and compared with GenBank^®^ reference sequences using BLASTn (blast.ncbi.nlm.nih.gov). The representative sequences were deposited in GenBank^®^.

Chromatograms were visually inspected for unresolved nucleotides. Sequences of 367 bp were then aligned and trimmed from primers using MEGA7 and the MUSCLE algorithm. *Schistocephalus solidus* (GenBank: OP586600) was used as an outgroup. The best-fit substitution model (HKY+G) was selected using JModelTest2, and a Bayesian Inference phylogenetic tree was constructed with the BEAST v2.7.7 package. Chain convergence and tree prior values were evaluated with Tracer v1.7.2. The consensus tree was estimated using TreeAnnotator and visualized in FigTree v1.4.4.

## Results

**Figure 1:**
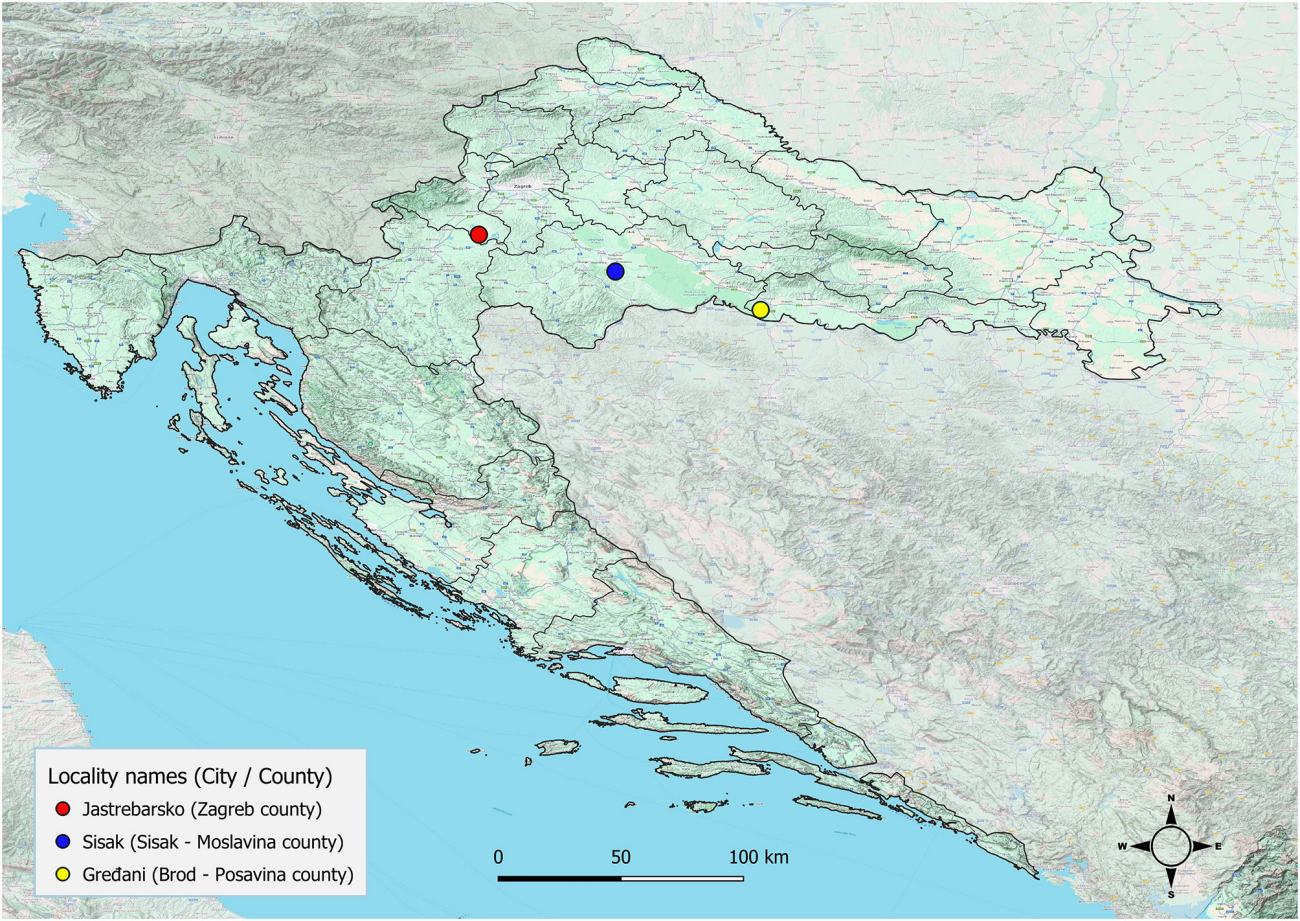
Geographic locations in Croatia where *Spirometra mansoni* was detected in. Figure was created with QGIS.

**Figure 2:**
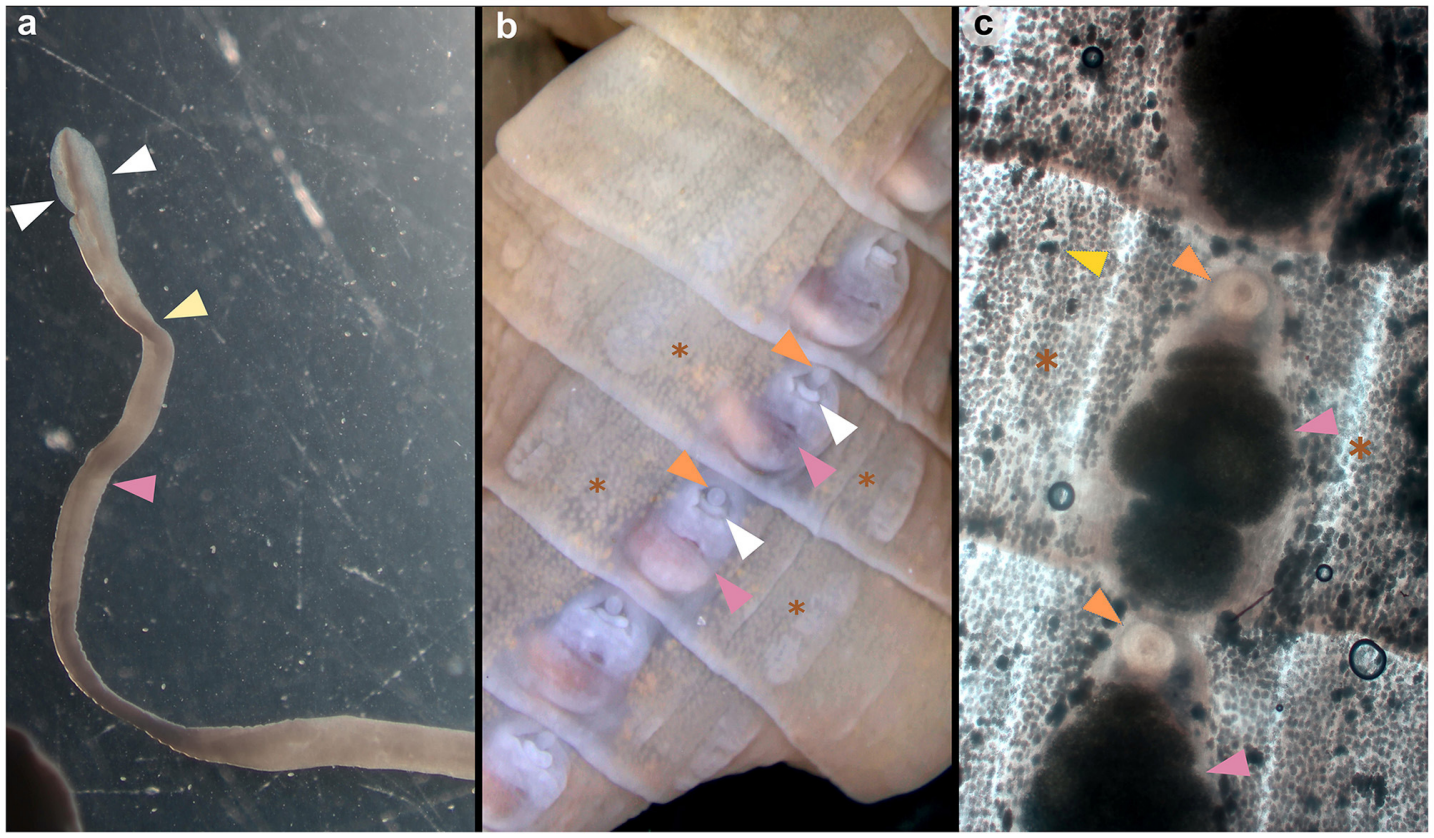
Morphological assessment of the scolex and proglottids of *Spirometra mansoni* collected from the golden jackal (*Canis aureus*) in Croatia. **(a)** Elongated scolex of an adult showing two bothria (marked with white triangles), the neck (yellow triangle) and the beginning of the strobila (pink triangle). **(b)** Mature proglottid with evident genital pore (marked with an orange triangle), uterine pore (white triangle), coiled uterus (pink triangle) and vitelline glands (brown asterisk). **(c)** Mature proglottid showing the genital pore (orange triangle), coiled uterus filled with eggs (pink triangle), testis (yellow triangle) and vitelline glands (brown asterisk).

Adult cestodes were identified in the small intestines of three golden jackals (1.5%) originating from three different locations in the central part of Continental Croatia (Figure 1). The Spirometra-positive animals were collected in Jastrebarsko (GPS: 45.662651, 15.679982) on September 5, 2022, in Gredani (GPS: 45.204596, 17.157740) on January 31, 2025, and in Sisak (GPS: 45.506042, 16.405342) on December 6, 2023. The morphological characteristics of the cestodes included an unarmed scolex with well-developed bothria and a narrow neck separating the scolex from the immature proglottids (Figure 2a). Under stereomicroscopy, both immature and mature proglottids were observed (Figures 2b, c). The proglottids contained multiple testes and vitelline glands in the lateral fields, with genital and uterine pores located medially. The genital pore was anterior to the uterine pore, and the uterus showed several convolutions, features consistent with *S. mansoni* description (7). The presence of a single cestode with distinct morphological characteristics was confirmed in each animal.

**Figure 3:**
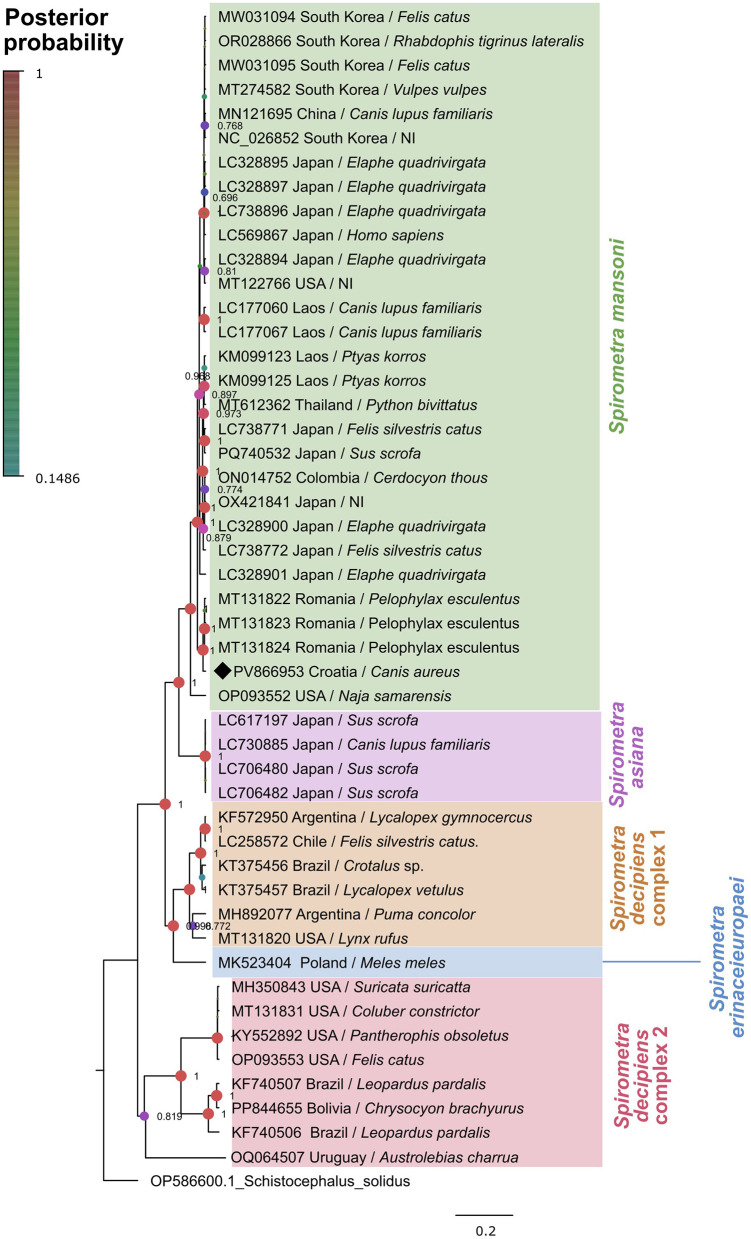
Phylogenetic analysis of *cox1* sequences of *Spirometra* spp. (1,567 bp) from different hosts and geographical regions. Bayesian inference phylogenetic tree showing *Spirometra* spp. in different color blocks. The *Spirometra mansoni* sequence obtained this study is marked with a black diamond.

Molecular analysis of the mitochondrial genes confirmed the identification of *S. mansoni*, with all three tested specimens exhibiting identical nucleotide sequences. The *cox1* sequences showed 98.53% similarity with *S. mansoni* from *Pelophylax esculentus* (GenBank accession numbers MT131822 and MT131823), with 100% query coverage. Molecular analysis of the *nad1* gene revealed sequence similarities ranging from 95.09% to 96.32% with *S. erinaceieuropaei* (GenBank accession numbers OM935779 and OM935776), 95.30% with *S. ranarum* (MH298844), and 95.71% with *S. decipiens* (MN121695), all with 100% query coverage. Phylogenetic reconstruction of the *cox1* using Bayesian inference placed the Croatian sequences within the *S. mansoni* clade with a posterior probability value of 1.0, indicating strong statistical support (Figure 3). This clade included sequences from diverse geographical regions including Asia and Europe (Romania), confirming the wide distribution and relatively low inter-population variability of this species.

## Discussion

This study presents the first confirmed molecular identification of *S. mansoni* in golden jackals in Croatia and, to our knowledge, in Europe. Gherman and Mihalca (22) cite a case report from 1889 describing the necropsy finding of *S. mansoni* (syn. *Bothriocephalus mansoni*) in a golden jackal in Italy (23). However, a golden jackal originated from Egypt and was imported into Italy. Furthermore, the identification was based solely on subcutaneous larvae and lacks morphological or molecular confirmation. The case is not included in the more recent and comprehensive reviews by Kuchta et al. (4, 7), and therefore, we consider the 1889 report to be unreliable. Although sparganosis and its etiological agents have been well documented in Asia and Africa, reports from Europe remain scarce and are largely based on morphological identification alone (4, 7). To date, *S. mansoni* has been molecularly confirmed in Europe only in water frogs from Romania (4). Our findings support recent recommendations advocating the integration of molecular and morphological methods to accurately delineate species boundaries and improve knowledge of *Spirometra* distribution (4, 7). Previous evidence of *Spirometra* spp. in Croatia was based solely on morphological re-examination of parasite eggs found in gray wolf feces (4, 24), without molecular confirmation. Since *Spirometra* spp. eggs are indistinguishable among species and morphology of mature proglottids alone is insufficient for reliable species confirmation, molecular typing remains essential for accurate identification at specie level (4, 7). Thus, this study represents the first genetically verified record of *S. mansoni* in a wild canid in the country.

The identification of *S. mansoni* in a free-ranging wild carnivore in Europe is unexpected, as *S. erinaceieuropaei* has previously been considered the only endemic species on the continent (4). The sequences clustered within the *S. mansoni* clade with strong posterior support and showed haplotype identity with strains from Asia and Romania, raising the question about possible introduction pathways, host mobility, and underestimated zoonotic potential in the region. The lack of nucleotide variation among the three Croatian isolates indicates haplotype homogeneity within the local population. This may reflect either a highly conserved lineage or a recent introduction of *S. mansoni* into the region; however, the limited sample size precludes definitive conclusions. Therefore, more extensive sampling including other carnivore species and populations from neighboring countries is required to better assess local genetic diversity.

The golden jackal is a highly adaptable and increasingly widespread carnivore in Europe (16, 25). Its opportunistic feeding habits, including possible predation or scavenging on amphibians and reptiles (26), which are known second intermediate hosts of *Spirometra*, make it a suitable definitive host. Central Croatia, a transitional ecological zone between the Pannonian lowlands and the Dinaric Alps (where the jackals were sampled), is rich in natural water sources, and high biodiversity, creating optimal conditions for complex parasitic life cycles. The role of golden jackal as both a reservoir and a bridge host for emerging parasites in Europe was already emphasized (22, 27). The expansion of jackals from Croatia eastward (toward Bulgaria) and westward (toward Italy) has also been genetically confirmed (28). These factors may facilitate the local maintenance and potential spread of *S. mansoni* to other suitable wild reservoirs. Like other mesocarnivores, golden jackals successfully inhabit human-dominated habitats, where they rely on human-subsidized food sources, including scavenging and occasionally preying on domestic animals (29, 30). This behavioral flexibility facilitates frequent contact with both humans and domestic animals. In Costa Rica, the possible circulation of *S. mansoni* between wild reservoirs (such as *Canis latrans*) and domestic animals (cats and dogs) has been described (8). Although the transmission pathways of *S. mansoni* are not yet fully understood, the presence of infected carnivores in close proximity to human settlements may carry potential public health implications that warrant further investigation.

Although *S. mansoni* has been associated with human infections in other regions, its zoonotic potential in Europe remains unclear. The presence of this parasite in an expanding and synanthropic carnivore such as the jackal warrants heightened awareness and additional surveillance. Our study highlights the importance of wildlife in maintaining the life cycle of zoonotic cestodes and the utility of molecular tools for reliable species identification. In addition to the complete *cox1* gene sequence, we generated and deposited a new *nad1* sequence of *Spirometra mansoni* in GenBank^®^. While previous sequences of *nad1* associated with this species exist (although labeled under different names such as *S. erinaceieuropaei, S. ranarum*, or *S. decipiens*), our sequence contributes to the growing molecular reference dataset of *S. mansoni*, which is crucial for accurate phylogenetic comparisons and lineage resolution (4, 31). Future studies should include a broader range of potential definitive and intermediate hosts within the region, such as amphibians (e.g., frogs), reptiles (e.g., snakes), and domestic carnivores (e.g., cats), to elucidate the full transmission network of *Spirometra* spp. and assess possible spillover risks to humans.

## Conclusions

This study provides the first molecular confirmation of *S. mansoni* in the golden jackal in Croatia and possibly Europe. The detection of this zoonotic cestode in a free-ranging wild canid expands current knowledge of *Spirometra* host range and distribution on the continent. These findings support the need for increased public and veterinary awareness, as well as targeted surveillance and education of professionals regarding the zoonotic potential of *Spirometra* spp. in Europe.

## Data Availability

The datasets presented in this study can be found in online repositories. The names of the repository/repositories and accession number(s) can be found in the article/supplementary material.
